# Institutional Notes and News

**Published:** 1910-03-19

**Authors:** 


### Institutional Notes and News.

**British Lying-in Hospital, Endell Street.**

The annual general meeting of the British Lying-in Hospital was held on March 8, Mr. Charles E. Farmer, the Chairman of the Board of Management, presiding. In proposing the adoption of the report for 1909, the chairman pointed out that the number of in-patients exceeded by 18 those of 1908, and the number of out-patients was less by 23, the former being 535 and the latter 542. One maternal death occurred during the year, the patient having been admitted in a collapsed condition. The excess of expenditure over income was £349 13s. 11d., against £673 2s. 4½d. in 1908, the expenditure side of the account showing considerable reductions over the previous year, which is highly satisfactory. The cost of each in-patient has been reduced by 13s. 9d. A sub-committee has lately inquired into the cost of working the hospital, and has reported that, though in larger institutions more favourable results may be obtained by reason of the larger contracts, with the possible exception of two items mentioned, all proper economy is exercised. But, Mr. Farmer explained, it seems almost impossible to carry on the work with the income received. A form of bequest has been sent out with the reports this year, which, he hopes, may obtain for the charity more legacies in the future. There are fifteen members of the Ladies' Committee, and they do their work well, and the convalescent cottage at Barnet is worked by them. They find patients in need of rest and fresh air and send them down there to recruit. The chairman deeply regretted to report the loss by death of one of the vice-presidents, the Marquess of Ripon, who has been a supporter of the hospital for many years.

**Royal Free Hospital.**

The recent meeting of the Royal Free Hospital, Gray's Inn Road, disclosed a satisfactory state of affairs, which in its financial bearings is no doubt partly due to the gift of £4,000 which the hospital received last year from the King's Fund. In the absence of the Earl of Sandwich the chair was taken by Sir Edwin Durning-Lawrence, and the meeting was informed that the hospital is face to face with a problem that is already exercising the thoughts of the management. That problem is the reconstruction of the out-patient department. At the present time, it will be remembered, the existing accommodation for out-patients is practically in the basement of the building, and neither the patients nor the medical staff has fair opportunities of receiving or administering adequate treatment. It is clear from the position of this department that any change must include rebuilding. The question of the site is therefore one of paramount importance, if only because the acquiring of new land in this part of London is, as was pointed out, a very costly business. One of the speakers said he believed that land might be acquired if the money to pay for it were forthcoming. The only other alternative which presents itself is in some way to make use of the open space which forms the quadrangle of the hospital. Any scheme of this sort, which, of course, would be far cheaper than buying fresh land, would have to overcome, however, the criticism that it would interfere with the present air and light enjoyed by the wards which surround the quadrangle. The in-patients for the year numbered 2,225, the out-patients 36,892, and the average number of beds occupied was 144. The income from all sources was £19,516, which included £6,726 from legacies, reducing the total ordinary income to £11,410. The total expenditure was £17,851, of which £17,360 was ordinary for maintenance. It will therefore be seen to what an unfortunate extent the Royal Free Hospital has to rely upon legacies for purposes of income. The ordinary receipts fall very far short of the ordinary expenditure.

**Sheffield Royal Infirmary.**

The new building at the Sheffield Royal Infirmary, which is soon to be finished, will contain 180 beds, divided between six wards; two operation theatres, and a flat roof for the open-air treatment. Towards the £40,000 which these improvements are costing, £31,000 has been promised or received as the result of the Duke of Norfolk's appeal. The report of the recent annual meeting was proposed by the Lord Mayor, who pointed out that their income was not decreasing, but neither is the population of Sheffield. In the 18 months since the last report 4,998 in-patients have been received and 33,991 out-patients, while the average number of occupied beds has been 236. The income for last year was £15,018, and the expenditure £17,682—a reduction, explained Mr. John Marshall (chairman of the board), owing to the policy of economy they were pursuing.

**London Homœopathic Hospital.**

The chief feature at the recent annual meeting of the London Homœopathic Hospital, Great Ormond Street, was a resolution asking the governors and subscribers attending it to empower the board of management to withdraw £6,000 from the reserve fund to build a nurses' home, and £5,000 to complete the new Sir Henry Tyler wing according to the original plan. This was agreed to, it being pointed out by Lord Cawdor, who presided, that a conditional gift of £5,000 was promised from Lord Dysart if the remainder were raised by the end of the year. This together with Mrs. Evans' legacy, the money for which has now been received, will not make the withdrawal from the reserve fund the serious thing that it otherwise would be. The patients treated last year numbered 1,063 and 11,620 in the in- and out-patient departments respectively.

**Year's Work at Cumberland Infirmary.**

In July 1908 an ophthalmic department was established at the Cumberland Infirmary, and that extension has made its influence felt on the annual report, which presents figures which are a record in the history of the institution. There were 1,150 in-patients, nearly 100 more than in 1908, and 4,098 out-patients, of whom 580 came for ophthalmic treatment to the new department of the hospital. Owing to the reduction of the average stay of each in-patient to 28 days, the increased number of patients treated has had what at first seems the curious result of reducing the daily average number of occupied beds by four or five, the average for the past year being 88. The Agnes Moore Fund, which assists the Convalescent Home at Silloth, has done capital service in helping 57 patients to receive the completion of their cure by convalescent treatment. There is no doubt that some of the most useful work in the whole of hospital treatment is done by this after-cure or convalescent home system, which is springing up now all over the country. The total income from all sources was £5,514, and at this point it may be noticed that the earnings of the private nursing staff amounted to £369, of which the estimated profit is £157, or about £50 less than in 1908. Against this income must be set an expenditure of £5,903, leaving a deficit on the year of nearly £400, and a total deficit against the hospital of £715. It is worth adding that £245 was contributed by patients, and that Miss M. R. Huddart's legacy of £519 was placed to the building fund. A new item in the expenditure accounts is one for electricity. The x-ray apparatus, which has worked well for seven years, has now to be replaced, and an up-to-date plant is to be installed costing £100. Meanwhile the extension scheme sanctioned two years ago has been progressing—the new boiler-house, laundry and kitchen blocks, and nurses' home are now completed, the home, indeed, being occupied just before Christmas. The new west wing, comprising a male surgical ward of 12 beds, and a children's ward, on the first floor, of 24 cots has still to be constructed. The Bishop of Carlisle, who, as President, took the chair at the annual meeting, in commenting on the falling-off of the annual subscriptions, said that it would be a disaster if the threatened municipalisation of the hospitals ever became an accomplished fact. Dr. Barnes, who has been Chairman of the Committee of Management for six years, during which period great changes have taken place, and great improvements been made in the financial position of the infirmary, has tendered his resignation, to everyone's regret.

**Bourton Cottage Hospital.**

Bourton-on-the-Water possesses a cottage hospital in a very satisfactory financial state. The annual report was able to state that for 1909 the receipts were £31 15s. more than in the previous year, and that the expenditure for the corresponding period had decreased by £48. It should be remarked here that £44 was subscribed by patients. Forty-two in-patients, in exactly equal proportions men and women, were admitted during the year, and the average number of days they stayed was 44. The hospital, however, still needs new surgical instruments, and this spring some expense will be incurred in painting the outside of the building. Special thanks were recorded to Miss Whiteside, the matron, for her work, so there is no doubt that the first half-century of its life, which is completed this year, will be crowned with success on every side.

**Guy's Hospital.**

The conversion of the old Front Surgery into an Actino-therapeutic Department is practically completed, and this will be opened for the treatment of patients very shortly. The new surgical x-ray room at Out-Patients is also now complete and in use. A scheme has been approved by the Governors for the conversion of the present light department on the top floor into a new suite of operating theatres. The plans provide for three new theatres, lighted from the top and sides, with galleries for onlookers, and smaller rooms adjoining for the administration of anæsthetics. The latter is a very desirable feature, and its absence from the existing theatres is frequently commented upon by visitors. In addition there are to be sterilisation and preparation rooms, and a room for the pathological examination of specimens.

**Carlisle Dispensary.**

The Carlisle Dispensary, which is perhaps the oldest charity in the city, having been established in 1782, was just able to square its accounts for the year. But the annual report was compelled to admit a slight decrease in the annual subscriptions. These receipts amounted to £421, which, when added to a balance of £139 from the previous year, totalled £560. The expenditure was £417, so this year the dispensary can hand on a slightly increased balance, namely £142, to the credit of the institution. There were 1,816 house patients and 1,823 persons treated in the out-patient department, making a total of 3,639. The death of the resident medical officer, Dr. Stephenson, who had only been appointed in 1907, was a matter which the report particularly regretted to record. His successor is Dr. Mackinnon, of Edinburgh. Dr. Sedgwick remarked at the meeting at which the report was presented that since he had been at the dispensary he had never seen a case of its abuse by the poor, for whom its work was invaluable.

**Infirmary and Children's Hospital, Kidderminster.**

Owing to certain criticisms alleging extravagance and waste on the part of the management of the Infirmary and Children's Hospital, Kidderminster, Dr. Walter Moore, its President, emphasised certain portions of the annual report at a recent Court of Governors. He said that the report of the Committee of Inquiry into the question of expenditure has dissipated the fears that some people entertained. Last year was a year on which the infirmary might congratulate itself. By means of the fund, started by him, to free the infirmary from debt, together with the profits of the year's working, not only have all liabilities been cancelled, but there is a balance to the credit of the hospital. Dr. Moore summed up the work of his year's presidence, and the work to be done by Major Ellis Talbot, the succeeding President, by saying "it was my duty to clear off the debt, it will be his to see that the infirmary does not run into debt again." The resignation of Miss Barling, the matron, has been accepted with regret by everyone at Kidderminster Hospital.

**Royal Victoria Infirmary, Newcastle.**

A serious state of affairs is revealed by the recent annual report of the Royal Victoria Infirmary, Newcastle. These are the facts. The 430 beds which the infirmary possesses are practically always full. Indeed, Dr. Drummond, who has been physician at the infirmary for 30 years, went so far as to say that the work had been advancing by leaps and bounds to a fabulous extent. Last year there were 7,872 in-patients, and no fewer than 83,295 out-patients, in each case double what the numbers were ten years ago. These figures, Dr. Drummond remarked, explained why the staff had induced the House Committee to inquire into the question of admission, and to see whether or not the gates of the infirmary had not been opened a trifle too wide. In his opinion the causes of this vast extension of the hospital's work were a growing population, the popularity of the infirmary, the increase in the three special departments of ear, eye, and throat, largely due to the working of the Act for the medical inspection of school children. He was afraid lest in time the infirmary should fall beneath the weight of work fastened upon it. The expenditure last year was £35,540, and the income £31,242. The Lord Mayor, who presided at the annual Court of Governors, said he was struck by the disparity between the workmen's contributions and those of ordinary subscribers. In five years the working classes had trebled their gifts, which last year reached the splendid sum of £16,531. He, with others present, cried shame on the colliery owners, whose subscriptions last year were only £175! The same story which applied to the colliers as compared with the colliery owners, applied to local workmen and the firms by whom they were employed, for in only a few cases were the workmen's subscriptions supplemented by an equal sum from the employers. The Subscriptions' Committee is considering the difficult question of drawing some distinction between contributing and non-contributing areas and giving priority of admission to those cases which come from districts which subscribe.

